# A national survey on depressive and anxiety disorders in Afghanistan: A highly traumatized population

**DOI:** 10.1186/s12888-021-03273-4

**Published:** 2021-06-22

**Authors:** V. Kovess-Masfety, Katherine Keyes, Elie Karam, Ajmal Sabawoon, Bashir Ahmad Sarwari

**Affiliations:** 1 Conseil Santé, Clichy, France; 2 Department of Psychiatry, McGill University, Montreal, Canada; 3 Université de Paris, LPPS, Boulogne-Billancourt, France; 4 Mailman School of Public Health, Columbia University, New York, NY USA; 5 Institute for Development, Research, Advocacy & Applied Care (IDRAAC), Beirut, Lebanon; 6 Dept. of Psychiatry & Clinical Psychology, St. George Hospital University Medical Center University of Balamand, Faculty of Medicine, Beirut, Lebanon; 7 Governance Institute of Afghanistan (GI-A), Kabul, Afghanistan; 8 Kabul University of Medical Sciences (KUMS), Kabul, Afghanistan; 9 Dept of Mental Health & Substance Abuse, Primary Health Care Directorate, Ministry of Public Health, Kabul, Afghanistan

**Keywords:** Mental health, Epidemiology, Survey, Trauma, Transcultural, Risk factors

## Abstract

**Background:**

This survey attempts to measure at a national level, exposures to major traumas and the prevalence of common mental health disorders in a low-income dangerous country, highly affected by conflicts: Afghanistan.

**Methods:**

Trans-sectional probability survey in general population by multistage sampling in 8 provinces, represented nationwide: 4445 adults (4433 weighted),15 years or older, 81% participation rate. Face to face interviews used specific scales for measuring lifetime exposure (LEC 5 Life Events Checklist) and Post Traumatic Stress Disorder (PTSD Check List), a diagnostic standardized interview: Composite International Diagnostic Interview (Short Form) for.

Major Depressive Episode and Generalized Anxiety Disorder, plus scales for suicidal thoughts and attempts and psychological distress (MH5 and RE from SF36).

**Results:**

52.62% of the population is illiterate, 84,61% of the women do not have any source of income; 70.92% of the population lives in rural areas, 60.62% are below 35 years, 80% lives in very dangerous areas. 64.67% of the Afghan population had personally experienced at least one traumatic event; 78.48% had witnessed one such event. 60.77% of the sample experienced collective violence in relation to war and 48.76% reported four or more events; this very much differs across regions and levels of danger; women are less at risk for trauma except sexual violence, 35 years and above are more at risk than younger. 12-month PTSD prevalence reaches a high rate: 5.34% as MDE 11,71%, whereas GAD 2.78%; suicidal thoughts 2.26%, lifetime suicidal attempts 3.50% are close to reported in other countries. Women have more risk for PTSD (0R = 1.93) and suicidal behaviours (attempts OR = 1.92) than men; the number of events increases risk for MDE, PTSD and suicidal attempts, whereas education is protective. Exposure to different war events produced different mental health effects. People suffering from PTSD have higher risk to report 12-months suicidal ideations and lifetime suicidal attempts.

**Conclusion:**

Our findings highlight the need to map the extent and the types of mental disorders post conflict; this would help maximise the help to be offered in guiding proper choice of interventions, including education.

**Supplementary Information:**

The online version contains supplementary material available at 10.1186/s12888-021-03273-4.

## Background

Afghanistan has gone through phases of external invasion and civil strife for more than three decades, and most Afghans have been exposed to these events [1]. Persistent war has resulted in the destruction of Afghanistan’s economic, social and cultural infrastructures. It has forced Afghans to live through challenging conditions, such as frequently being bereaved, having their land and properties damaged, being forcefully obliged to move internally or externally, and being imprisoned and tortured in neighboring countries as well [2]. Recurring natural disasters, such as drought and earthquakes, also had an impact on the mental health and well-being of the Afghan population [3]. A wealth of evidence from both high income and low income countries indicates that war and political violence are associated with high rates of mental health disorders and are associated with the risk of long-term mental health problems [4], yet the impact of war and disaster on mental health in Afghanistan is insufficiently researched.

To date, several epidemiological studies have been conducted, though limitations in the sampling frames and time periods argue for additional research. In 2000 a survey in two Afghan regions plus a refugee camp in Pakistan, comparing Taliban controlled versus non controlled zones, reported high rates of women’s clinical depression and suicidal thoughts in the controlled zones [5]. Further, psychological distress was assessed in a national survey in 2002 [6] and a regional survey in 2003 in the Nangarhar province [7]. However, the screening tools used by both studies—the Hopkins Symptom Check List and Hopkins Trauma Questionnaire—although well-known and largely used, were not designed to distinguish between mental disorders and reactions to severe environmental stress. Thus, whether the symptoms reported represented clinical disorders or expected response to severe stress remains difficult to distinguish [8]. Additionally, the rates of measured disorders do not reflect the experience of clinicians, especially a high rate of reported PTSD, which may be indicative of non-representative sampling or other methodological problems [9]. None of these studies, with the exception of the last cited, were recent and none extensively measured the diverse mental health disorders following DSM classifications criteria and risk associate factors.

The present study, which is unique as it is being done on a national basis, covered quasi-inaccessible regions, some of them being extremely dangerous, and aims to measure exposure to major traumas, the prevalence of the most common mental health disorders including Post Traumatic Stress Disorder PTSD (Blevins et al.), Major Depressive Episode (MDE), Generalised Anxiety disorder (GAD), suicidality and the relationships between trauma exposure levels and mental health disorders controlling for the main socio-demographic mental health determinants.

## Methodology

### Population

The household survey was implemented in each of the eight regions of Afghanistan in order to represent the whole country: 1) Eastern; 2) South Eastern; 3) Southern; 4) Western; 5) North Western; 6) North Eastern; 7) Central Kabul; 8) Central Bamiyan. A multi-stage stratified cluster sampling method was applied: in each region, two provinces were randomly selected totaling 16 provinces out of the 34 provinces of the country, then the Central Statistical Organization (CSO), after random sampling of clusters for each selected province, provided the list and maps of 320 randomized clusters. At the same time, a thirty cluster sampling method, based on WHO recommendation was also applied. The balance of rural and urban settings was considered together with the region sizes and counted in proportion. Considering security concerns, ~ 10 clusters had to be replaced with the nearby cluster in the same district or the next district. The surveyors were then required to complete questionnaires for 14 households randomly selected in each cluster. In the household, a randomised adult selection was based on Kish selection before starting the interview. It involved Afghan males and females at least 15 years old and resident, and those who had given consent to participate in the study. The sample size required by region was 542, that is, 4336 persons for the country as a whole. A consent form had to be read aloud and accepted for each selected person before completion; those who did not accept were excluded.

Participation rate was on average 90% for household (93 to 86% depending on the region) plus 90% for selected individuals (100 to 50% depending on the region), which corresponded to 81% total response rate.

The Afghan population is relatively young; correspondingly, more than half of the sample was below 35 years old and 4.47% were 65 or older. However, the sample slightly differs from the total Afghan population as described by the recent official estimate (Estimated Population of Afghanistan 2107–2018, Central Statistics Organisation Islamic, Republic of Afghanistan, 2017), in that there were more people over 35 years and more women than expected based on population census estimates. Since gender and age composition differ across regions, sample weights were estimated for representation of the general population.

The share of urban areas was around 10 to 50% among selected clusters, except Kabul Province, where urban areas represent around 70% of the clusters including Kabul city. For the total sample 73.10% of the people live in rural areas, the remaining 26.90% in urban areas as compared to the total Afghan population, which corresponds to national estimates.

**Fig. 1:**
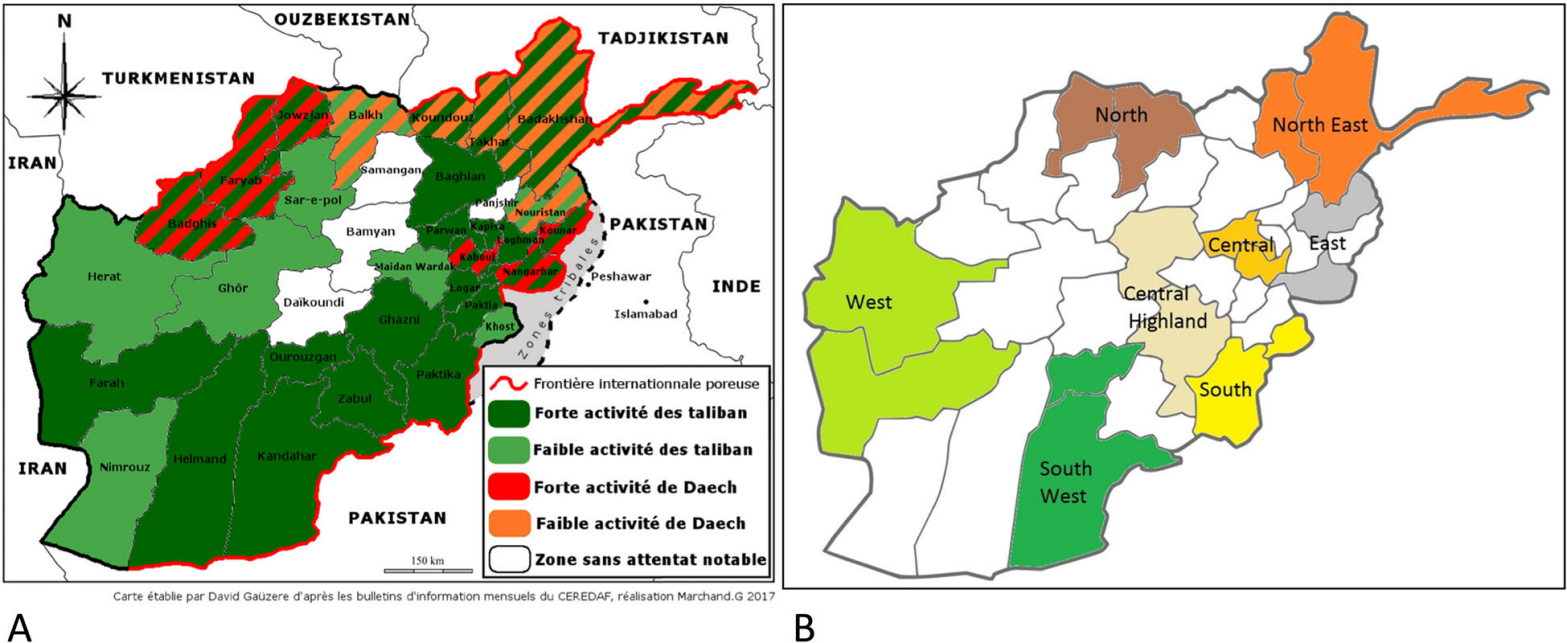
**A** Levels of danger by province based upon Taliban and Daech terrorist activities in 2017 as recorded by the French NGO CEREDAF (“fort”=high, “faible”=low). **B** Map of Afghanistan showing the regions that were selected

To estimate exposure to dangerous situations, we attributed to each of the 16 provinces a score in accordance with the scores for Taliban or ISIL activities evaluated by a French NGO: “Centre d’Etudes et de Recherches Documentaires sur l’Afghanistan” (CEREDAF) (Fig. 1). Based on these estimates, 3.7% of individuals live in no activity/danger areas, and 29.35% in mildly active/dangerous areas (which experience mild activity from ISIL, Taliban or both); these individuals were combined into a “low level” category. Approximately, 29.37% liv in highly dangerous area that experiences either.

Taliban or ISIL strong activity levels; 37.58% % live in very high danger areas that experience both Taliban and ISIL strong activity levels, or one strong and the other at a mild level.

### Instruments

The questionnaire collected pertinent socio-demographic information: gender, age, educational level, occupation (position and sector), income and ethnicity.

Psychological distress and impairment due to mental health problems were measured by SF36 which contained two subscales scales on mental health dimensions: one which measures transient symptoms of anxiety and depression which constitutes psychological distress, (Amowitz) and the other which measures the extent to which mental health problems interfere with daily tasks (Emotional Role) for which we applied the recommended threshold [10].

The Life Event Checklist five LEC5 [11] was used in order to assess lifetime exposure to 16 events, separately recorded as personally experienced or witnessed, known to potentially result in PTSD or distress together with the PTSD Checklist for DSM-5, and a 20-item self-report measure that assesses the 20 DSM-5 symptoms of PTSD [12, 13]. These symptoms were: reliving of what happened, alertness, avoidance of feelings or memories, extensive suspiciousness; all symptoms at an intensity that impairs the life of the person. Diagnostic’s algorithms were assigned based on question responses corresponding to DSM-5 criteria. Events were questioned on a lifetime period, while impairment was reported in the last 12-months, allowing for PTSD 12-month prevalence reporting. For comparison purposes, we clustered the prevalence of LEC-5 events as proposed by the WMH (World Mental Health Initiative), a set of surveys pooling together 24 countries, among them low income and countries at war, into categories obtained by factor analysis [14]. In addition we built a “number of events” variable coded as: none, one to three, four and more traumatic events either self-experienced or witnessed, in accordance with the number of events carrying PTSD risk as found in the WMH set of surveys. (Elie G [15].).

Major depressive episodes and Generalised Anxiety Disorders were measured with the CIDI Short Form. The Composite International Diagnostic Interview (CIDI) is a fully structured diagnostic interview, aimed to assess disorders, using the definitions and criteria of the (DSM). It is built to be used by lay interviewers, who collect information on closed standardised questions; then an algorithm reproduces the diagnoses criteria. It has been validated in the WMH surveys, which covered many developed and developing countries [16–18]. CIDI SF, its short form, has been developed by Kessler [19] et al. for the National Canadian survey and, since then, used in different countries and contexts, among them in Europe; validation has been established in many languages [20]. The suicidal behaviour section and the disability scales of Sheehan have been added to each diagnosis to assess functional impairment in work/schools, social and family life.

A 5-day training provided instruction on survey method, cluster identification, fraction process at the cluster, a comprehensive and detailed review of questionnaires, plus one day in the field testing the questionnaires. In total the data collection process mobilised 64 teams of two individuals (one male and one female staff). The number of teams varied for each province, based on the number of clusters, with 9 teams for Kabul and 1 team for Farah province. There was one supervisor for each province to manage the administrative aspects and coordinate the data collection process. Interviews were given in Dari or Pashto according to the language spoken in the selected household. Language/ethnic specificities in the different provinces were taken into consideration by recruiting appropriate interviewers. Questionnaires were read aloud to participants and efforts were made to respect privacy as much as possible.

### Statistics

Analyses were done with STATA 15.1. Since the composition of the observed sample differed from the expectations as assessed by Afghan statistics, tables are presented weighted on gender and age compositions, and logistic regressions allowed evaluation of the main risk factors.

## Results

52.62% of the weighted individual sample completed no formal education and did not have any reading skills, 3.5% did not complete primary school, 6.4% completed primary, 8.47%secondary, 18.1% college and 7.85% university. 53.5% of the sample declared no income, including as much as 84.61% of women and 22.62% of men. 13.68% of the sample declared to work in agriculture or animal husbandry, 13.8% as laborer, while 9.11% are salaried, 3.7% in business or trading. Income was very much linked to the type of employment: those in agriculture, farming or as laborer earned an average of 7200 AFS (100$) whereas those in business earn 12,982 AFS (200$). Female disadvantage persisted for the few who had an income: 42% in the lowest income category versus 16% of men. Urban people belonged to higher income groups than rural people. On the total weighted sample, 27.55% were Tajik, 47.82% were Pashtu, 11.4% Hazara, 6.58% Uzbek, 6.65% another ethnicity (for 0.08% the information was missing) (Table 1). As expected, ethnicities were very different across regions.

**Table 1:** Sample description

		Total	Men	Women	
	N weighted^a^	4433	2225	2208	
Education					
	Never attended school	52.62	37.37	67.92	*P*=0.0000
	Less than primary school	3.50	4.43	2.56	
	Primary school	6.38	7.67	5.08	
	Secondary school	8.47	9.98	6.96	
	High school	18.2	25.65	10.72	
	University	7.85	11.11	4.59	
	Other	2.98	3.80	2.16	
Occupation					
	Service/ Salaried	9.11	14.43	3.75	*P*=0.0000
	Business/Trading	3.66	6.61	0.70	
	Worker	13.8	23.42	4.12	
	Agriculture/Animal husbandry	12.67	24.01	3.27	
	No source of Income	53.5	22.62	84.61	
	Other	5.31	8.27	2.34	
Income Afs					
	N	n=1726			
	<=3000	20.51	15.98	43.91	*P*=0.0000
	>3000 >=6000	30.11	31.66	22.09	
	>6000<=10000	30.40	32.60	19.00	
	>10000	18.97	19.74	14.97	
Residence					
	rural	73.1	75.25	70.92	*P*=0.0028
	urban	26.9	24.74	29.07	
Level of Danger					
	low/moderate	21.41	22.94	19.87	*P*=0.0000
	Medium High	41.00	34.40	47.66	
	High	37.58	42.66	32.46	
Ethnicity					
	Tajik	27.55	26.11	29.00	*P*=0.0000
	Pashtun	47.82	47.59	48.05	
	Hazara	11.40	9.96	12.85	
	Uzbek	6.58	8.27	4.87	
	Other	6.65	8.06	5.20	
Age					
	<35	60.44	60.27	60.62	*p*=0.8132
	>=35	39.55	39.73	39.38	
Marital Status	Never married	29.02	33.76	24.24	*P*=0.0000
	Married	67.24	65.06	69.43	
	Widowed/div or sep	3.74	1.17	6.33	

^a^N non weighted 4 446 : 1876 Males, 2570 Females

### Traumatic events

For each trauma listed, the LEC 5 assessed the level of implication: the event could be experienced by individual, or witnessed; these two experiences could, and in war or terrorism context usually did, occur together but they could also happen independently.

The population of Afghanistan was highly exposed to traumatic events: 64.67% had personally experienced at least one traumatic event with or without witnessing somebody else’s trauma; among them 7.68% had experienced a trauma without witnessing traumas happening to others; 78.48% had witnessed one such event, with or without personal a trauma experience, among them 21.39% have only witnessed traumatic events without personal trauma; 57,36% have both experienced and witnessed trauma. Ultimately 86.16% of the population had either personally experienced or witnessed at least one traumatic event in his or her life. (Table 2).

**Table 2:** Type of event weighted % of persons

	Central Kabul	South	East	South West	West	North	Central High.	North East	Total exp or Witnessed	Total experienced by the individual
**Collective Violence**	**55.17**	**68.68**	**76.36**	**68.33**	**64.71**	**32.63**	**69.98**	**50.09**	**60.77**	**27.58**
*Fire or explosion*	43.70	55.62	6.02	50.59	45.19	19.42	56.66	33.98	45.68	10.89
*Assault with a weapon*	14.93	37.88	21.40	26.92	24.68	08.73	20.30	16.81	21.28	5.65
*Combat or exposure to a war zone*	22.33	41.38	49.09	42.94	33.24	13.73	38.5	27.02	33.44	16.35
*Captivity*	10.63	24.2	19.71	14.57	26.99	08.77	26.34	16.08	18.58	5.50
**Caused/Witnessed Harm**	**11.72**	**26.34**	**18.5**	**17.11**	**18.43**	**04.53**	**10.14**	**05.32**	**13.95**	**1.02**
*Sudden violent death*	09.64	22.4	17.99	15.56	15.25	04.02	08.41	05.02	12.21	**NA**
*Serious injury. harm or death you cause to some body else*	02.71	10.90	01.04	04.21	04.6	00.47	03.31	02.52	03.66	**1.02**
**Inter-personal Violence**	**34.04**	**62.15**	**41.23**	**52.89**	**49.19**	**22.6**	**49.73**	**31.48**	**42.70**	**25.01**
*Physical assault*	34.04	62.15	41.23	52.89	49.19	22.6	49.73	31.48	42.70	25.01
**Sexual Violence**	**1.83**	**14.76**	**1.20**	**7.51**	**2.72**	**0.52**	**0.85**	**4.06**	**3.99**	**2,02**
*Sexual assault*	1.43	10.47	0.22	5.00	1.76	0.52	0.85	3.88	2.87	0.57
**Accidental Injury**	**86.56**	**78.68**	**90.53**	**69.44**	**78.38**	**59.70**	**81.04**	**87.15**	**79.23**	**50.94**
*Natural disaster*	*77.54*	*64.61*	*83.43*	*49.07*	*60.14*	*49.52*	*71.08*	*84.6*	*67.90*	*40.08*
*Transportation accident*	*36.26*	*54.51*	*58.51*	*42.54*	*43.63*	*24.41*	*47.51*	*30.00*	*42.22*	*13.53*
*Serious accident at work*	*18.05*	*31.76*	*31.84*	*30.51*	*25.40*	*02.37*	*17.66*	*07.07*	*20.46*	*6.73*
*Exposure to toxic substances*	*05.71*	*14.26*	*04.22*	*12.36*	*17.81*	*03.92*	*12.97*	*05.76*	*09.63*	*2.31*
*Life threatening illness or injury*	*16.86*	*30.39*	*24.41*	*15.55*	*28.59*	*03.97*	*11.50*	*06.60*	*17.33*	*5.28*
*Sudden accidental death*	*14.71*	*24.67*	*21.34*	*23.37*	*23.41*	*09.32*	*12.40*	*07.40*	*17.01*	*17.01*
**Other**	***15.45***	***13.83***	***14.27***	***04.77***	***28.80***	***3.44***	***12.09***	***5.34***	***12.51***	**7.35**
**Any Event Exp or witnessed**	**89.12**	**88.04**	**95.55**	**83.12**	**84.84**	**67.33**	**87.42**	**92.37**	**86.13**	
Any event experienced	82.95	63.61	74.97	55.68	68.65	46.85	63.99	57.65	64.67	**64.67**
any event witnessed	73.84	79.95	89.39	79.99	81.01	49.07	83.09	89.71	78.48	

Exposure to events was different across region and each statistical comparison test was highly significant (*P* < 0.001). Collective violence such as assault with a weapon, combat or exposure to a war zone, was very frequent: 60.77% of the sample experienced collective violence, including 27.58% as a personal experience, 55.1% as witness. Large interregional differences were apparent; 76.36% in the East region to 32.63% in the North. Interpersonal violence was reported by 42.7% of the population; 25% of the population were physically assaulted; 33% witnessed the event. Large interregional differences were apparent; 12.17% in the North to 37.19% in the South. Sexual violence was relatively infrequently reported, and rape was reported by 0.97% of women, 0.18% of men; witnessing it was nearly equally reported by men (2.61%) and women (2.21%) (*P* = 0.40).

Finally, accidental injury was reported by 79.23% of the population: 50.94% as a victim, 69.37% as a witness; except in the North region, and to a lesser extend to the South West, this type of trauma was remarkably frequent and reported by up to 90% of the population. Since Afghanistan is prone to earthquakes, natural disaster was the most frequent such event: 67.8% of the population had experienced it, and exposure varied according to the region. Transportation accidents were also quite frequent: 42.18% of the population: 13.54% as victims and 37.19% as witnesses, again with large regional variations. (Table 2).

Victims of trauma experienced on average 2.17 traumas and those who witnessed traumas witnessed an average of3.92 events. As a result, when grouped together, those who experienced or witnessed an event, the average number is 4.16. 14.33% of the population did not report any traumatic event, 36.90% reported one to three and 48.76% four or more.

### Mental health prevalence

Overall point prevalence of psychological distress was 47.12% and substantial impairment due to mental health was 39.44%. 12-month PTSD prevalence rate was 5.34%, GAD 2,78% and the 12-month MDE was11.71%; 12-month suicidal thought prevalence was 2.26% with a wide range: 0.08 to 4.94% across region. Lifetime suicidal attempts were 3.50% ranging from 1.10 to 7.60%and prevalence of lifetime suicidal thoughts was 8.23% ranging from 3.72 to 14.81%.

Women suffer more than men from psychological distress, impairment due to mental health, PTSD and suicidal behaviours and suicidal thoughts; whereas, no such differences were found for major depressive episode and generalised anxiety disorders. (Table 3).

**Table 3:** Mental health outcomes by region

Region	Last Month High Psychological Distress	Last Month Impairment of role due to MH	Major Depressive Episode 12 months	PTSD 12 months	GAD 12 months	Suicidal Thought Lifetime	Suicidal Thought 12m**	Suicidal Attempt Lifetime **
N=4432 weighted								
Central Kabul	48.64	43.66	6.03	6.15	5.35	12.96	4.18	5.92
South	58.68	55.85	6.12	10.08	3.37	14.81	4.94	7.60
East	37.77	37.00	8.04	3.58	1.79	5.71	1.73	2.80
South West	50.4	32.91	2.21	5.05	1.16	6.9	2.26	2.98
West	53.48	53.65	7.54	6.24	4.12	9.91	2.24	3.69
North	34.34	31.63	4.06	3.88	0.37	1.66	0.78	1.10
Central High Land	55.29	29.14	2.40	4.90	4.81	10.09	0.08	2.65
North East	38.65	30.73	1.88	3.07	0.91	3.72	1.34	1.42
**Overall Prevalence** *CI 95%*	**47.12** *45.54-48.70*	**39.44** *37.93-40.97*	**4.86** *4.23-5.57*	**5.34** *4.71-6.04*	**2.78** *2.33-3.32*	**8.23** *7.41-9.13*	**2.26** *1.84-2.77*	**3.50** *2.97-4.11*
Male	**37.58**	**21.99**	5.07	**4.11**	2.49	**5.41**	**1.78**	**2.28**
Female	**46.68**	**26.62**	4.65	**6.57**	3.07	**9.10**	**2.74**	**4.58**

*yes & do not know; In bold *p*<0.0001

**N=4365

### Exposure to trauma and mental health

Logistic regressions in Table 4 showed that for each type of event, gender is a main predictor: women have less than half the probability of being exposed (OR from 0.32 to 0.57) than men, except for sexual violence, where it was the opposite (OR = 1.49). Subjects whose age was 35 years or older, were more exposed, and significantly so, to collective violence, and caused or witnessed body harm type. Being married was protective against sexual violence (OR = 0.80) as compared to be single (Table 4). Compared to Tajiks, Pashtuns were more exposed to collective violence (OR = 1.71) whereas those outside the main ethnicities (“other”) seemed protected against it (OR = 0.66). Others and Hazaras seemed protected from accidental violence (0R = 0.51 and 0.67). Living in a dangerous area, increased the probability of exposure to cause or witness of bodily harm and sexual violence; for accidental violence only the highest level of danger is significant. To live in a rural area as compared to urban was a risk for sexual violence: OR = 2.09. Location was a main predictor of exposure: compared to central/Kabul, North and North East regions seemed protective for each type of event, whereas the remaining regions seemed more at risk. Sexual violence followed the same pattern except for North East, where that risk seemed higher.

**Table 4:** Exposure to trauma logistic regression (weighted)

	N=4364	Any violent Event	Collective violence	Cause/witness Body Harm	Inter personal violence	Sexual violence	Accidental violence
**Gender**	Male	ref					
	Female	**0.46**	**0.32**	**0.55**	**0.44**	**1.49**	**0.57**
**Ethnicity**	Tajik	ref					
	Pashtun	**1.52**	**1.71**	1.16	1.48	1.81	1.27
	Hazara	0.81	0.81	0.82	1.08	1.14	**0.67**
	Uzbek	1.14	1.30	0.87	1.25	1.82	1.04
	Other	**0.65**	**0.66**	0.96	0.78	1.34	**0.51**
**Region**	Central Kabul	ref					
	South	1.00	1.02	**4.01**	**2.40**	**9.58**	0.82
	East	**1.80**	**1.60**	1.17	0.85	**0.30**	1.18
	South West	0.78	1.19	**2.28**	**2.16**	**3.77**	0.58
	West	1.09	1.38	**3.23**	**1.69**	2.57	1.04
	North	**0.39**	**0.33**	**0.57**	**0.51**	**0.42**	**0.41**
	Central High Land	1.54	**2.06**	1.44	**1.85**	0.59	1.58
	North East	1.09	**0.59**	**0.32**	**0.68**	**1.35**	0.76
**Education**	<Primary	ref					
	prim or second	0.90	0.84	0.96	0.94	0.47	0.83
	high or univ	1.24	1.06	1.22	0.93	1.16	1.28
**Level of danger**	Low/mild	ref					
	High	1.10	1.10	**1.39**	0.84	**1.62**	1.00
	Very High	**2.04**	1.03	**2.53**	1.02	**3.11**	**2.30**
**Residence**	Rural /Urban	1.07	0.98	1.26	1.17	**2.09**	1.03
**Age**	=>35 years/<35	1.06	**1.43**	**1.38**	0.93	1.23	1.08
**Marital statut**	Married/no	0.89	1.08	1.16	**0.80**	1.53	0.85

Exposure was determined by individual experience: type of events and number, and by the surrounding conditions: living in a specific region or in a dangerous area as defined by the NGO. We have developed three models to evaluate the role of individual versus environmental stressors. 1) a logistic regression evaluating the effect of each type of event and their numbers, on the diverse mental health outcomes, controlling for socio-demographics (gender, age, education, rural /urban residence and ethnicity.) 2) adding the region 3) adding the area’s level of danger. We have presented this last model in Table 5.

**Table 5:** Mental health outcomes determinants , logistic regression (weighted)

N=4204		Major Dep Episode 12m	PTSD 12m	Suicide Attempt LT	Suicidal Thoughts LT	MH5 Past month	RE Past month
**Gender**	Female/ Male (ref)	1.21	**1.93**	**2.43**	**1.92**	**1.64**	**1.35**
**Education**	*< Primary*						
	Pri/Secondary	1.24	0.90	0.94	0.96	0.84	**0.80**
	High school /University	0.75	0.78	**0.57**	**0.60**	**0.69**	**0.80**
Age	>=35/<35 years	1.25	**1.51**	0.79	0.88	**1.79**	**1.95**
**Ethnicity**	*Tajik*						
	Pashtun	1.32	0.95	1.14	**1.62**	**1.33**	0.97
	Hazara	*0.44*	0.66	*0.42*	1.07	**0.50**	*1.32*
	Uzbek	0.83	1.57	1.06	0.74	1.11	0.85
	Other	1.29	1.54	**2.54**	**2.43**	**1.46**	1.01
**Areas of residence**	Rural/ Urban (ref)	0.82	1.39	1.05	1.06	1.02	0.95
**Type of event**	Collective violence	0.91	1.29	1.14	1.14	**1.38**	*1.23*
	Cause/witness Body Harm	**1.87**	**2.84**	*1.47*	*1.36*	**1.38**	**1.44**
	Inter personal violence	1.37	1.15	**2.17**	**1.63**	**1.24**	1.16
	Sexual violence	**2.11**	**1.60**	**2.51**	**2.09**	**2.52**	**2.12**
	Accidental violence	0.63	0.82	0.93	1.06	0.93	1.18
**Exposure**	*none*						
	1 to 4 events	**5.67**	**6.63**	**3.94**	1.10	1.09	0.98
	>=4 events	**11.24**	**10.80**	**6.75**	0.88	1.41	1.46
**Level of Danger**	*Low/mild*						
	High	0.89	**2.41**	0.90	0.78	**1.28**	**0.66**
	Very High	0.84	1.90	1.62	1.52	**1.91**	1.00

Controlled for region; Bold *p*<0.05 Italic *p*<0.10

For MDE the 3 models indicated similar results: three types of events increase the risk: cause or witness of bodily harm, interpersonal and sexual violence; the number of events dramatically increased the risk, the highest level of danger carried its own risk in addition to age and gender effects; to live in the West (OR = 1.94) increased the risk and in North East decreased it (OR = 0.31) as compared to Central/Kabul region and to belong to the “other “ethnic group increased the risk (OR = 1.45).

Lifetime suicidal attempts had a slightly different pattern: to be a woman increased the risk significantly: 0R = 2.43, the number of events had as important an effect as the type of event; however, if sexual violence was a predictor for MDE and suicidal attempts, interpersonal violence was significant: OR = 2.17. Lifetime suicidal attempts were the only mental health outcome for which ethnicity seemed to have an effect: to belong to a different ethnicity than the main Afghan ethnic groups increased that risk (OR = 2.54). The three models brought similar results on the event’s roles but there was an important regional effect: in five of the seven other regions that risk was much lower than in the Central/Kabul region; adding level of danger did not have specific effect. Lifetime suicidal thoughts followed the same pattern, except for the exposure effects which did not show up.

Psychological distress is very sensitive to each of the factors, except the number of events, whereas level of danger has an important effect; reported impairment due to mental health was quite similar to psychological distress except for the level of danger. Regions had significant effect for both of these indicators.

The number of events had a lower effect than for the MDE and suicidal attempts: only the 4 and greater increased the risk: OR = 1.89; noteworthy is that there were huge regional effects: each region except one carried a much lower risk than, the Central Kabul region. To add the level of danger had no effect.

PTSD risk factors included gender and age; two types of event constantly increased that risk: cause/witness bodily harm and sexual violence; the number of events consistently had an important effect and level of danger did not overlap region: high level of danger carried its own risk together with some regions. Some risk factors for GAD differed from the other mental health problems: life in a rural area (OR = 2.61) and interpersonal violence (OR = 2.89), whereas the number of events did not seem to be a risk.

## Discussion

In order to compare the Afghan rates with international data, we used the WMH surveys clustering of events which covers a total of 24 countries, among them countries where violent conflicts happened. The six clusters are presented in Table 2.

Collective violence trauma cluster related to war was much higher in the Afghan sample than in the pooled WMH countries: 27,51% as a personal experience, 60.77% as witness and/or personal experience: versus 9.40% as witness or personal experience in WMH [14].

Among these collective violence traumas: 10.89% in person; 45.68% as witness and /or in person of the Afghan sample were exposed to a fire or explosion as compared to 4% in the WMH pooled samples; 5.50% of the Afghani sample reported having been in captivity or kidnapped versus 1.1% in the WMH sample. Participation in combat or exposure to a war zone was also higher in the Afghan sample: 16,35%personally, 33.44% as witness versus 6.4% who have reported living in a war or terror zone in the WMH pool [14]. Of course, in the WMH country sample, these exposures varied across countries: in Lebanon as compared to USA the OR for collective violence was 20.6, 4.0 in Northern Ireland, 3.8 in Nigeria, so the Afghan rates corresponded to countries at war or enduring terrorism. For example, in certain Afghan regions 49 to 41% of the population have been exposed to a war zone as compared to 55,2% in the Lebanese study (E. G [21].).

The interpersonal violence cluster consisted of being physically assaulted: 25% of the Afghan sample experienced it in person and 42.7% either witnessed or endured it, as compared to 17% in WMH.

Some specific traumatic events seemed less frequent than in the WMH countries such as sexual violence: 0.57% of the Afghan sample (0.97% for women) reported sexual assault, versus 9% in WMH (among them 3.2% victims of rape); to be noted is that most of the WMH countries have much lower rates than USA which is the reference country: except New Zealand, each other country had significant lower risks and some countries,mostly in Europe, had much lower risks: OR = 0.2 or 0.3 [14]. In addition, the sexual violence risk is far from equal in the different Afghani regions: in the South, sexual assault reached 2.58% (4.82% for women); 10.47% for witness and/or sexual assault.

In the accidental injury cluster: transportation accidents were equal in both samples: 13.53% versus 13.53% whereas natural disaster was much more frequent in the Afghan sample than in WMH: 42.7% of the population was involved in such trauma, as compared to 7.7% of the WMH pooled sample.

As in the WMH country samples, to be a woman carried a lower risk for each type of violence, except for sexual violence, where they are more at risk than men.

The 12-month PTSD rate is very high 5.34%: in comparison to the WMH surveys where the highest level was reached by Northern Ireland: 3.8%; Lebanon was at 1.9%. The average number of events for those who experienced or witnessed an event is 4.16, which is considered as a high risk for PTSD (Elie G [15].). In addition, in the WMH study which combined data from many countries, the PTSD risk was set up at four or more events as a threshold where cases of PTSD have greater impairment and morbidity. Consequently, these cases may be severe and impairing. As reported, women are more at risk than men and the older more than the young. Educational level is not protective, and the diverse ethnicities did not differ in their risks; the personal experiences of traumas carry a very important risk independently of the level of danger of the surrounding.

The prevalence of 12-month MDE is also very high 11,71%, as compared to rates reported in some WMH countries: twice the Lebanon rate: 6.6%, higher than the USA rate: 9.6% or Ukraine: 9.1%. Comorbidity with Major Depressive Disorder is high: a person suffering from PTSD has nine times more risk of suffering from depression as well OR = 9.41.

Curiously, PTSD diagnosis was not familiar to psychiatrists in Afghanistan: when we were conducting our validity studies on cases, the psychiatrists did not find any PTSD cases in their out-patient clientele, whether depressive, anxious or cases with substance use disorders. It could be that traumas are so frequently experienced by the population, that to have symptoms such as described by PTSD did not cause mental health consultation, whereas sadness, loss of pleasure and the other depressive symptoms were considered as abnormal. In fact, in another part of the survey, focused on seeking help for mental health problems: “consequences of trauma events”, which covers a larger scope than PTSD symptoms, is mentioned by 12,51%, whereas sadness is 65,46% and anxiety 27% [22].

Lifetime suicidal thoughts are comparable to what is reported in Europe, at least in the highest rate countries such as Northern Ireland, France, Germany, Portugal and Belgium. In each of these countries female rates are higher, as in Afghanistan [23]. Concerning the twelve-month rate of ideation, the Afghan rate: 2.26% is close to the 2.2% found in the WMH developing countries group [24].

The comorbidity of suicidal behaviours with PTSD is very high: people suffering from PTSD have a 9.6 higher risk of reporting 12-month suicidal ideations; 7.5 times higher risks for lifetime suicidal attempts. Therefore, it seems that the people exposed to trauma have the highest risk for suicidal behaviours as previously reported [25, 26]. However, in this survey, we do not have any information of what “causes” the suicidal behaviors. An increase of suicidal behavior has been described in Lebanon in relation to war events, mainly through the presence of mental health disorders, which carries such risk as major depressive episode (E. G [27].).

Some type of events such as collective violence and interpersonal and sexual violence that are frequently observed in a war situation, carried specific risk for MDE and GAD as it has been reported in the Lebanese study. Also, the cumulative effect of events is the same in both surveys: the risk of having PTSD at four events or more is identical (OR = 10) as well as the risk of developing MDE (OR = 3.32) (Elie G [15].).

Limitations of the study should be considered. To conduct a national survey in Afghanistan was an enormous challenge; the sample is as representative as possible under such circumstances. In very dangerous areas the clusters have to be replaced; in one of the provinces the participation rate was 50% only. Half of home interviews were done in the presence of children who could be adolescents or adults, mostly parents or “other adults”, rarely spouse. For sexual violence, certainly under reported, we have compared rates for interviews done alone with other attending controlling for respondent gender, but did not find any differences, however the OR for being alone versus not: 1,56 (95% CI 0.98–2.5) was close to significance (*p* = 0.063). Despite careful choices of interviewers according to gender and ethnicities, some other traumas may also have been underreported, such as inter-family violence.

In addition, questions had to be read aloud, and might have been misunderstood; for 10 out of 16 provinces, we added a question for the interviewer in order to have his/her opinion on how well the respondent understood the questions: 46.71% said excellent, 39.10% good, 8.62% fair, 1.74% poor and 3,86% did not complete the question, mainly in the West region: 12.94% did not complete, 3.74% were quoted as poor.

## Conclusions

Afghanistan is a multifaceted country: exposure and its mental health consequences largely varied by regions and ethnicities and this is an important element to consider in evaluating the mental health statute of the Afghan population. Personal exposure to traumatic events and to multiple traumatic events was by far the most important mental health determinant. Then gender, age, education level and level of danger of the environment mediated the mental health risks. PTSD was frequent in contrast to the other disorders such as major depressive disorders or generalized anxiety, which were at the rates that are usually reported in the literature in developed countries without conflicts. The PTSD prevalence is similar to what was reported in countries exposed to war, such as Lebanon and the prevalence of major depressive episode is huge.

As regards the disability resulting from Mental Health Disorders, close to one Afghan out of two is suffering from psychological distress and, is impaired in his or her role because of mental health problems. Individual’s multi exposure is a major risk and takes over the global environment level of danger except for PTSD where both risks remain independent. However, people seemed more conscious of the secondary disorders such as depression than of the direct trauma defined. To prevent the secondary effects of PTSD may be then a major public health issue.

Addressing the mental health needs of people exposed to disasters is well known by now and should be automatically included in any assistance programs, including international aid. An important issue to remember is the specificity of the mental health help needed in each war or disaster arena. Each war and each population has its own specificity. Based on our findings, an assessment of the types of mental health sequalae has to be conducted in a serious fashion to avoid a common pitfall, namely the application of a blanket treatment to all those exposed. This becomes acutely important if PTSD is the most prevalent disorder in the population at hand as we found in Afghanistan. This is important for many reasons; subjects with PTSD have a tendency to avoid consultation, especially in populations naive to mental health education: they want to avoid remembering the trauma. In addition, they do not know there is a treatment for it. This is less the case for GAD and depression where they might have acquaintances who sought treatment for these more established entities: PTSD is “new” to most people and always surprises the people affected by it, in addition to the fact that many might consider it a true weakness of character. Our findings highlight the need to map the extent and the types of mental disorders post (or intra) conflict; this would help maximise the help to be offered in guiding proper choice of interventions, including education. This would apply to any war, conflict or disaster.

## Supplementary Information

**Additional file 1.** On line table : Mental health problems model 1 socio demographic only logistic multivariate regression.

## Data Availability

The datasets generated and/or analysed during the current study are not publicly available due to properties rights of the funders and of the Afghan government. The data could be shared provided authorization of EU, the Afghan ministry of health and V.Kovess-Masfety.
