# Supplementary material for: A national survey on depressive and anxiety disorders in Afghanistan: A highly traumatized population
Source: BMC Psychiatry. 2021 Jun 22;21:314. doi: 10.1186/s12888-021-03273-4 (PMC8218387; doi:10.1186/s12888-021-03273-4)
Supplement: Supplementary file 1 — Additional file 1. On line table : Mental health problems model 1 socio demographic only logistic multivariate regression. [file 12888_2021_3273_MOESM1_ESM.docx]

| **On line table : Mental health problems model 1 socio demographic only logistic multivariate regression** | | | | | | | |
| --- | --- | --- | --- | --- | --- | --- | --- |
|  |  | **MH5** | **RE** | **Major Depressive Episode** | **PTSD** | **Suicidal thoughts LT** | **Suicide Attempt LT** |
|  |  |  |  |  |  |  |  |
| N=4390 |  |  |  |  |  |  |  |
| **Gender** | Female/Male | **1.35** | 1.09 | 0.90 | **1.53** | **1.47** | **1.89** |
| **Education** | < Primary |  |  | ref |  | ref | Ref |
|  | Pri/Secondary | 0.91 | 0.86 | 1.13 | 0.79 | 0.90 | 0.85 |
|  | High S  /University | **0.74** | 0.81 | 0.78 | 0.72 | **0.61** | 0.58 |
| **Marital statut** | Married/no | 0.97 | 1.10 | 1.14 | **0.66** | **0.74** | 1.04 |
| **Age** | >=35/<35 years | **1.78** | **1.77** | 1.27 | **2.03** | 0.91 | 0.81 |
| **Areas of residence** | Rural/Urban | 1.06 | 1.07 | 0.89 | **1.50** | 1.05 | 1.15 |
| **Ethnicity** | Tajik |  |  | ref | Ref | ref | Ref |
|  | Pashtun | **1.41** | 0.86 | *1.43* | 0.98 | **1.83** | **1.70** |
|  | Hazara | 0.95 | 0.97 | **0.33** | 0.58 | 0.60 | 0.52 |
|  | Uzbek | 0.80 | **0.65** | 0.79 | 1.84 | **0.44** | 1.29 |
|  | Other | 0.94 | 0.86 | 1.06 | 1.42 | 1.79 | 2.11 |
